# Current Grand Challenges in Allergy

**DOI:** 10.3389/falgy.2020.547654

**Published:** 2020-09-07

**Authors:** Nikolaos G. Papadopoulos

**Affiliations:** ^1^Division of Infection, Immunity and Respiratory Medicine, The University of Manchester, Manchester, United Kingdom; ^2^Allergy Department, Second Pediatric Clinic, National and Kapodistrian University of Athens, Athens, Greece

**Keywords:** allergy, asthma, rhinitis, eczema, hypersenitivity, hyperreactivity, immunity, allergen

## Allergy—What Is It?

The term “allergy” first appeared in 1906, coined by the Austrian pediatrician Clemens Von Pirquet, in an attempt to describe an unexpectedly exaggerated reaction to an innocuous stimulus. More than a century later, and despite extensive research and considerable understanding of the environmental triggers, biological pathways and clinical scenarios that fit into such concept, the limits of allergy remain controversial. The most formal proposal, sustained by the European Academy of Clinical Immunology, the American Academy of Allergy, Asthma and Immunology and the World Allergy Organization, suggests that allergy is an “*immune-mediated hypersensitivity*” (1). It is clearly stated that this approach includes but is wider than entities resulting from IgE-mediated pathophysiology. However, allergy is understood and used variably, in both wider and more narrow sense, by different lay and professional groups. Ambiguousness also infiltrates disease concepts within the field: archetypal diseases such as asthma, rhinitis, or food allergy display considerable variability in clinical presentation, epidemiology, or natural history, often termed *phenotypes*. Interestingly, diverse mechanisms (*endotypes*) may underpin the same or different phenotypes. To make things even more complicated (and interesting), coexistence of different allergic diseases in the same person is the norm rather than the exception, already starting from infancy, while the course of these conditions may follow different patterns. It is clear that a major challenge allergy researchers face is to classify clinical and mechanistic patterns within and between patients, in a way that will facilitate diagnosis, prognosis, and treatment.

While for many years polarized views of either a genetic background or an environmentally-driven disease, dominated the discourse, current thinking suggests that the observed variability may be the result of complex gene-environmental interactions. Therefore, the quest for a systematic characterization of possible configurations of these multidimensional dynamic interactions that lead to pathophysiological imbalance, is continuing (2).

## Re-Synthesizing Mechanistic Complexity: Immunity, Structure, Communication, Coexistence

The appreciation of the mechanisms of allergy is also a step toward understanding the wider picture of the human position within the biosphere. The principal functionary of self-definition and defense against “foreign” intrusion and/or internal damage, is *the immune system*. Not only numerous immune components are continuously scrutinized in relation to allergy development and expression, but we are increasingly approaching cells and mediators comprehensively, through omics and systems approaches (3). Moreover, we now know that “immune” functions are executed by many tissues beyond the traditional immune pathways (4). Structural cells and *the integrity of barriers* has attracted attention and will continue to be targeted as an area of high importance in delineating “in” from “out” (5). *Neural integration*, has only been slightly explored and is a good candidate for becoming the next hot thing, considering that several effective medications in allergy (such as beta-agonists or anticholinergics) directly target the nervous systems. With increasing presence and explanatory capacity, the *microbiome* is adding a new layer of complexity and will foreseeably continue to spearhead mechanistic studies and novel intervention attempts (6). A major challenge for this generation will be to harness the multitude of mechanistic information within a functional whole, forming and addressing unified hypotheses with impact in real life. Biological research is coming closer than ever to mathematics, demanding further *multidisciplinarity*.

## Recognizing Environmental Determinants and Supporting Environmental Sustainability

Hundreds of genes have been associated with a change of risk for allergy development, including genes that influence immune balance and regulation, structural characteristics of the target organs, as well as susceptibility to microbes. There is however little doubt that allergies are diseases strongly dependent of external exposures and a direct result of environmental changes that have taken place in the last one to two centuries. Once rare, allergies are now one of the major epidemics of our times, increasing in parallel to other chronic non-communicable inflammatory diseases, but only more rapidly and to an astonishing extent. Impressively and not yet explained, the presence of allergies varies hugely not only between countries but also between locations within a country (7). Environmental and *life-style management* is part of the armamentarium for allergy treatment. However, allergy epidemiology may also function as “a canary in a coal mine” to alert society for the impact environmental changes, including climate change, may have upon human health. To be even bolder, research into environmental determinants of allergy should spearhead the sustainability discourse and the need for healthier societies.

## Working Toward Prevention

During all of its history, a large part of allergy therapy has been based on avoidance of potential triggers and symptom relief. However, there is optimism that the epidemic and its associated suffering are preventable (8). Strong proof of principle studies support the feasibility of *allergy prevention*. Certain lifestyles have been consistently associated with low allergy risk. Recent human studies show that high allergen exposure diets early in life may prevent specific food allergy development. We still need some time before a universal “allergy vaccine” becomes available, but we're on it! A key requirement and direction is the focus on young children and pregnant women, considering that during these periods much of the tolerance development (and breaking) takes place.

## Incorporating Molecules in Everyday Practice

One of the major developments of the last decades in the field of allergy has been the advent of *molecular allergology*. Specific molecular structures within the allergens, often falling into distinct protein groups, have been identified as drivers of IgE sensitization and reaction; recognition and immune response against these structures can vary considerably between individuals, partly explaining the clinical diversity in allergy. Knowledge on allergenic molecules can increase diagnostic specificity and has potential for improved specific treatments (9). To date however, only a very small part of this potential has been released, partly due to regulatory hurdles in dealing with high complexity.

## Releasing the Full Capacity of Allergen Immunotherapy

*Allergen immunotherapy* is a unique treatment approach that has been part of the allergy field since its inception. Almost a century before the notion of “precision medicine” came at the forefront of medical philosophy, Noon and Freeman have demonstrated that treatment of an allergic disease is possible through gradual introduction of a targeted allergen. In conjunction with our widening new grasp of molecular allergens, this has the potential of generating truly personalized interventions. However, this type of approach cannot yet be digested by a regulatory framework requiring tens of millions of euros worth of standardization per unique molecule. Still, considerable novelty is been generated with new adjuvants, carriers, hypoallergenic molecules, preparations and routes of administration. It is a moving puzzle requiring solution, with potential for disease modification and a consequent effect in epidemiology and public health (10).

More recently, *oral immunotherapy* protocols targeting food allergy have been put forward and have center stage in regards to their usefulness and position in management. While these treatments are quite effective in desensitizing patients while under treatment, only a small proportion of cases develop long-term tolerance, while side-effects are frequent. The ongoing debate would hopefully lead to safe options, identify the patients who may benefit more and provide better understanding of the mechanisms involved.

## Positioning the New Precision Medicine in Allergy

Therapeutic monoclonal antibodies *(“biologics”*) are currently been introduced in the clinic as key interventions against severe forms of allergy, in the context of a “precision medicine” framework. Their main targets are currently IgE, IL5/eosinophils, and the IL4/IL13 pathway; efficacy has been proven, to a different extent and sometimes unexpected scope, in asthma, urticaria, atopic dermatitis and chronic rhinosinusitis with nasal polyps. Additional pathways are under scrutiny in clinical trials and the field is expected to expand further. Biologics have generated a lot of excitement, both for the prospect of their therapeutic efficacy, but also since they provide opportunities for improved understanding allergic inflammatory pathways in the human system. Considering the high cost of these agents and the competition for recruitment of severe patients in clinical trials, it is crucial to embrace this opportunity with prudence and inquisitiveness, as it may provide answers to endotypic classification and consequent further precision medicine options (11).

## Bringing Innovation to Real Life

In an increasingly cost-conscious world, precision targeting will also be necessary to define the acceptance of any new intervention by the payers. *Cost-efficacy* will most probably be incorporated in evaluating potential impact from very early on in research planning and even brilliant ideas will need to identify phenotypic niches in which they may be commercially viable.

However, although medications are globalized, as are most guidelines, the patient journey remains diverse between and sometimes even within geographical areas. In a world of international standards, a major challenge will be to *develop care pathways* accommodating knowledge of treatment efficacy and risk management within the framework of a particular health system and available resources (12). In this respect, technology, increasingly appearing as *e-health and m-health*, will certainly need to be harnessed (13).

**Figure 1:**
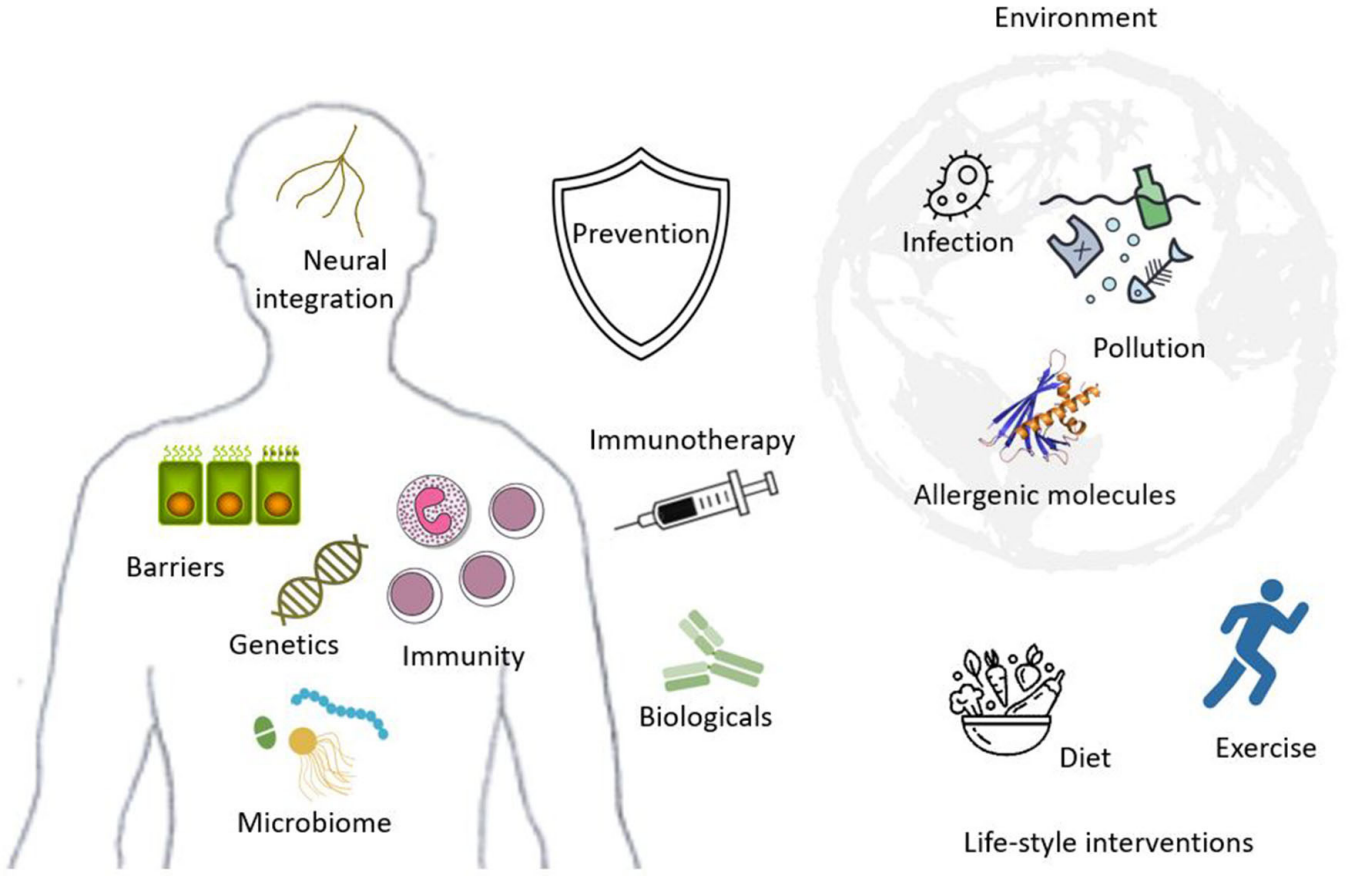
Exposure to a variety of environmental factors (upper right) including non-specific such as pollution or infection and specific allergenic molecules, influenced by life-style (lower right), including diet and activity, determine the framework of the current allergy epidemic. In genetically susceptible hosts (left), immunity, epithelial barriers, the microbiome and neural integration are affected, generating the clinical presentations of diverse allergic conditions. Allergen specific immunotherapy or manipulation of immune pathways through biologicals are developing treatments; however prevention remains the ultimate goal for allergy management.

In conclusion, allergy is a young, dynamically developing field. Understanding the ecological and mechanistic determinants and translating them into real-life interventions is ongoing and holds promise in tackling this public health epidemic, while revealing new aspects of our relationship with the environment (Figure 1).

## Author Contributions

The author confirms being the sole contributor of this work and has approved it for publication.

## Conflict of Interest

The author declares that the research was conducted in the absence of any commercial or financial relationships that could be construed as a potential conflict of interest.
